# Revision rhinoplasty: measurement of patient-reported outcomes and analysis of predictive factors

**DOI:** 10.1186/s40064-016-3166-5

**Published:** 2016-09-01

**Authors:** Ozan Luay Abbas

**Affiliations:** Department of Plastic, Reconstructive and Aesthetic Surgery, Faculty of Medicine, Ahi Evran University, 40000 Kırşehir, Turkey

**Keywords:** Revision, Rhinoplasty, Outcome, Satisfaction, Provided care, Preoperative information

## Abstract

**Background:**

Considering that revision rhinoplasty is one of the most difficult plastic surgical procedures, evaluating patient satisfaction is fundamental in order to determine success and identify variables that may affect the outcomes. Our first study objective was to determine satisfaction levels in revision patients and to compare results with those obtained in primary rhinoplasty patients. Second, we sought to identify factors that may influence the degree of satisfaction.

**Methods:**

Satisfaction was evaluated in 54 revision and 54 primary rhinoplasty patients using the rhinoplasty outcome evaluation questionnaire. To identify associated factors, patients were assessed for demographic characteristics, medical history, follow-up time, reason for revision, graft usage, the severity of nasal deformity, and satisfaction with the provided care and information given before the surgery.

**Results:**

All revision and primary rhinoplasty patients experienced improvements in satisfaction scores. Although the improvements were higher in primary rhinoplasty patients, the levels obtained in revision patients can be considered high. We found that young and male patients tend to have less satisfaction increment after the surgery. Patients who underwent revision for aesthetic reasons had higher improvements in satisfaction scores when compared to those patients who underwent revision for a combination of aesthetic and functional reasons. The improvement in satisfaction scores in patients who were satisfied with the information given before surgery was higher.

**Conclusion:**

Our data suggest that significant patient satisfaction is achieved after revision rhinoplasty and highlight the importance of the informed consent process when planning revision, especially on young and male patients.

**Level of evidence:**

III.

**Electronic supplementary material:**

The online version of this article (doi:10.1186/s40064-016-3166-5) contains supplementary material, which is available to authorized users.

## Background

Rhinoplasty is one of the most commonly performed procedures in plastic surgery practice. However, this surgery may also be perceived as one of the most difficult and unpredictable of all facial surgical procedures. This is because there are multiple dependent anatomical components and three-dimensional forces that have to be managed during the surgery. In addition, surgeons have to deal with both aesthetic and functional concerns of the patient at the same time. Consequently, it is not surprising that patient satisfaction rates after rhinoplasty are relatively low when compared with other facial cosmetic surgeries (Alsarraf 2000).

Up to this point of time, most scientific works about rhinoplasty offer objective parameters such as techniques, complications, anthropometric values, and so on (Bagal and Adamson 2002; Vuyk et al. 2000). Unfortunately, outcome studies for the evaluation of success represent a neglected area of research in this field (Rhee and Daramola 2012). Because traditional metrics such as mortality and morbidity mean very little in rhinoplasty, there is a need for evidence-based conclusions in order to assess the actual outcome. An important way to achieve progress in this gap lies in determining the degree of patient satisfaction after the surgery. In this context, many studies have been conducted to validate a questionnaire that evaluates patient satisfaction after rhinoplasty by assessing self-image and quality of life (McKiernan et al. 2001; Meningaud et al. 2008). Alsarraf et al. (2001) offered the rhinoplasty outcome evaluation (ROE) questionnaire for the assessment of patient satisfaction after rhinoplasty, with reliability, validity and internal consistency (Alsarraf 2000). This questionnaire takes into consideration the main factors that may determine patient satisfaction after rhinoplasty: the degree of satisfaction about nasal shape and function; the social, familial and professional acceptance; and the degree of self-confidence and desire to change nasal shape (Izu et al. 2012).

Revision rhinoplasty is generally more complex procedure than primary rhinoplasty because the tissues have scarred and been disrupted from the previous surgery. In addition, revision patients are generally stressed and traumatized from the previous surgery, which aids in complicating the psychological sides of an already complex task (Davis and Bublik 2012). Therefore, each subsequent surgery becomes more difficult, rendering perfection a difficult goal. These facts highlight the importance of proper patient selection in securing a good outcome and high satisfaction. For this reason, it is important to identify certain qualitative variables that may affect the degree of patient satisfaction after revision rhinoplasty.

To the best of our knowledge, there are hardly any studies in the literature investigating the outcomes of patients seeking revision rhinoplasty. Hellings and Nolst Trenite (2007) found that long-standing patient satisfaction can be achieved after revision rhinoplasty. This fact influenced the design of the current study and our search for knowledge about the outcomes of revision rhinoplasty and the underlying predictive factors. Since the previous study has evaluated the degree of satisfaction in revision patients only, our first study objective was to determine the levels of satisfaction in both revision and primary rhinoplasty patients in order to be able to make a comparison. Second, we sought to analyze several factors that may influence the degree of patient satisfaction after the revision such as the demographic characteristics of the patients, mental health issues, follow-up time, graft usage, indication for revision, the severity of nasal deformity, and satisfaction with the provided care and information given before the surgery.

## Patients and methods

### Patient recruitment

This study was approved by Ahi Evran University Institutional Review Board and Ethics Committee (Kırşehir, Turkey) and access to the patients’ data was granted. All procedures performed were in accordance with the ethical standards of the 1964 Helsinki declaration and its later amendments. Informed consent was given by all participants. Patients were selected from the author’s rhinoplasty database. 78 consecutive patients who underwent revision rhinoplasty between November 2012 and May 2014 were identified to be included in the study as the revision rhinoplasty group. Exclusion criteria included age less than 18, the presence of a congenital craniofacial anomaly and seeking revision for solely functional reasons. 71 Patients who fulfilled the inclusion criteria were invited by a phone call to visit the clinic and attend the study. 54 Patients accepted the invitation and agreed to participate in the study. The control group was composed of 54 primary rhinoplasty patients operated in the same time interval. All primary and revision rhinoplasties were performed by the author using the open approach. 3 (5 %) of the primary surgeries of the revision group had been performed by the author.

### Data collection methods

1. *Analysis of the medical records* The medical files of all patients who fulfilled the inclusion criteria were verified for demographic characteristics, medical histories, the indication of the revision and technical details about the surgery.
2. *Evaluation of patient satisfaction before and after rhinoplasty* The assessment of rhinoplasty-related patient satisfaction was performed using the ROE questionnaire (Additional file 1). The patients in both groups were asked to complete the ROE questionnaire twice during the same visit. The answers in the first questionnaire were based on the preoperative photographs (frontal, lateral and inferior views) of each patient in order to evaluate the degree of satisfaction in the preoperative setting. The answers in the second questionnaire assessed each patient’s actual outcome. This method is similar to previous studies published by other authors (Hellings and Nolst Trenite 2007; Arima et al. 2011, 2012). In the ROE questionnaire, patients were asked 6 questions about the degree of their satisfaction. Each question was answered on a scale ranging from 0 (the lowest satisfaction) to 4 (the higher satisfaction). The sum of the scores was divided by 24 and multiplied by 100, which leads to the final score. Higher scores were found to be associated with greater patient satisfaction with the outcomes.
3. *Evaluation of patient satisfaction with given information before the surgery* We evaluated patients’ satisfaction with the information given before the surgery using 6 questions with “yes” or “no” responses (Additional file 2). A patient was judged as “satisfied with the given information” if he/she have answered “yes” on more than 3 out of 6 questions (Ronnberg et al. 2007).
4. *Evaluation of patient satisfaction with provided care* The patients’ satisfaction with the provided care was assessed using 7 questions with “yes” or “no” responses (Additional file 3). A patient was judged as “satisfied with the provided care” if he/she have answered “yes” on more than 4 out of 7 questions (Ronnberg et al. 2007).
5. *Objective evaluation of nasal deformity* In order to determine the severity of nasal deformity objectively, two independent plastic surgeons not involved in the study scored the nasal shape of each patient using standard preoperative photographs. The frontal, lateral and inferior views were scored on a scale from 1 (great deformity) to 5 (no deformity). The total score was calculated, with a score of 15 showing perfect nasal shape. The consistency between two surgeons was evaluated with the Spearman correlation. The Spearman correlation coefficient was high (0.948), showing a remarkable degree of agreement between observers.

### Statistical analysis

Power analysis was performed to determine the optimal sample size. To compare two independent groups, the Mann–Whitney U test was used. Kruskal–Wallis test was used to compare multiple independent groups. Correlations between variables were evaluated using Spearman’s rho correlation coefficient. Data analyses were performed using the Number Cruncher Statistical System (Kaysville, Utah, USA). Results were considered statistically significant at *p* < 0.05.

## Results

### Patient characteristics

A total of 78 patients underwent revision rhinoplasty in our department between November 2012 and May 2014, of whom 54 (19 males and 35 females) fulfilled the inclusion criteria and agreed to participate in the study. The average age of the patients was 33.5 (range, 23–45). The control group was composed of 54 (21 males and 33 females) primary rhinoplasty patients with an average age of 30.5 (range, 19–46). The characteristics of both groups are given in Table 1.

**Table 1:** Patient characteristics

Demographic characteristic	Primary rhinoplasty n = 54	Revision rhinoplasty n = 54
Age, years ± SD (min–max)	30.5 ± 6 (19–46)	35.5 ± 6.5 (23–45)
Sex, no. (%)		
Female	33 (61)	35 (65)
Male	21 (39)	19 (35)
Marital status, no. (%)		
Single	24 (45)	19 (35)
Divorced	4 (7)	3 (6)
Married	26 (48)	32 (59)
Psychiatric history, no. (%)		
No	51 (94)	51 (95)
Yes	3 (6)	3 (5)
Follow-up, months ± SD (min–max)	15 ± 6 (6–26)	13 ± 5 (6–23)
Reason for revision, no. (%)		
Aesthetic only	–	30 (55)
Aesthetic + functional	–	24 (45)
Graft, no. (%)		
No	–	36 (66)
Costal cartilage	–	10 (19)
Conchal cartilage	–	8 (15)

### ROE questionnaire satisfaction scores

**Fig. 1:**
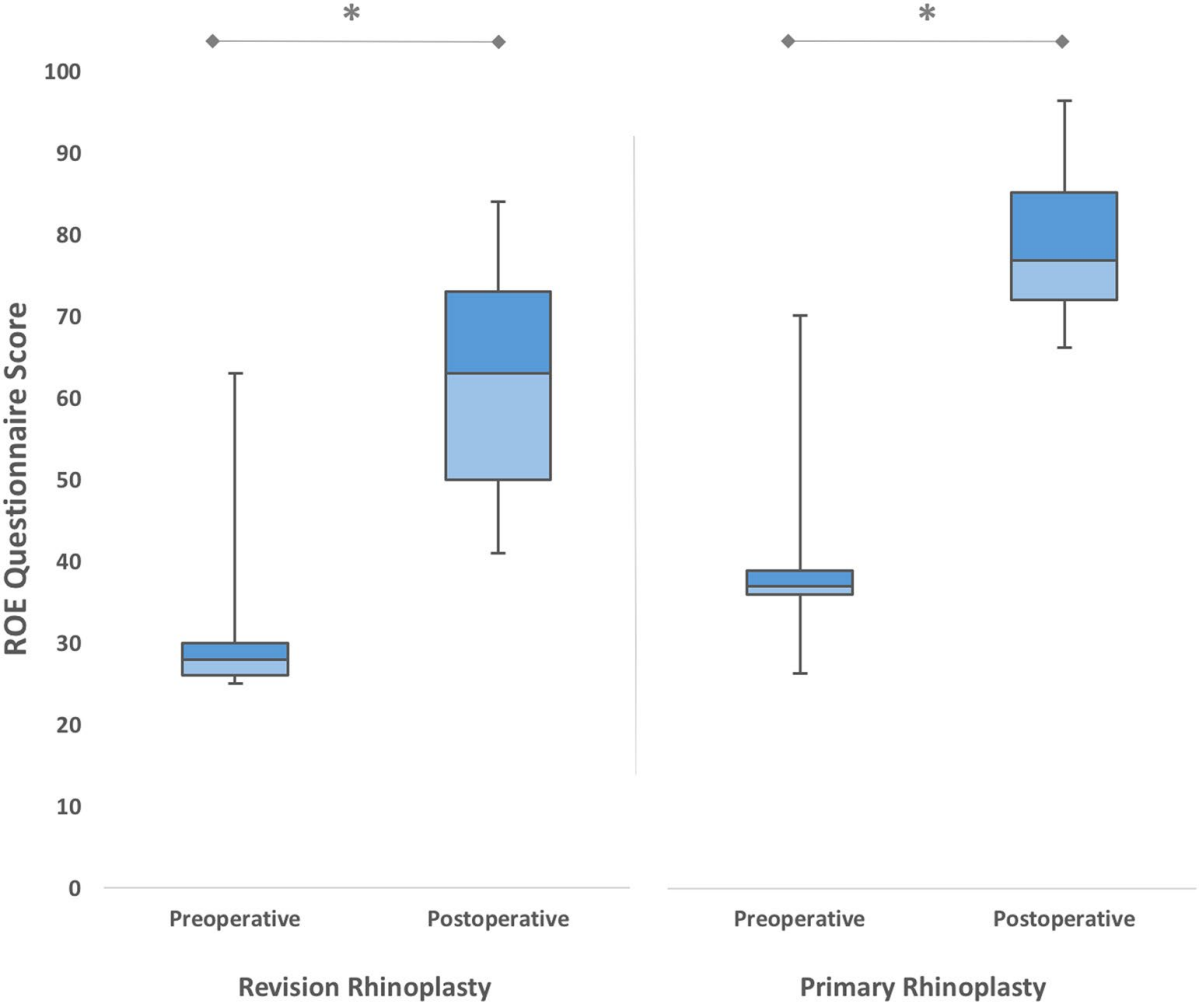
The mean preoperative and postoperative satisfaction scores of patients who underwent revision and primary rhinoplasty (*p < 0.001)

In both groups, all postoperative satisfaction scores were higher than those of the preoperative. In the revision rhinoplasty group, the mean preoperative satisfaction score was 32.81 ± 11.03, and postoperatively the mean increased to 62.40 ± 12.44. There was a difference between the mean scores in the preoperative and postoperative of 29.59 ± 9.81 (*p* < 0.001). On the other hand, the mean preoperative satisfaction score in primary rhinoplasty group was 40.51 ± 8.06, and postoperatively increased to 80.53 ± 8.90. The improvement in satisfaction scores (40.01 ± 7.66) was statistically significant (*p* < 0.001) (Fig. 1). The difference between two groups in means of improvements in satisfaction scores was statistically significant (*p* < 0.001).

### ROE questionnaire satisfaction scores in relation to patients’ characteristics

**Fig. 2:**
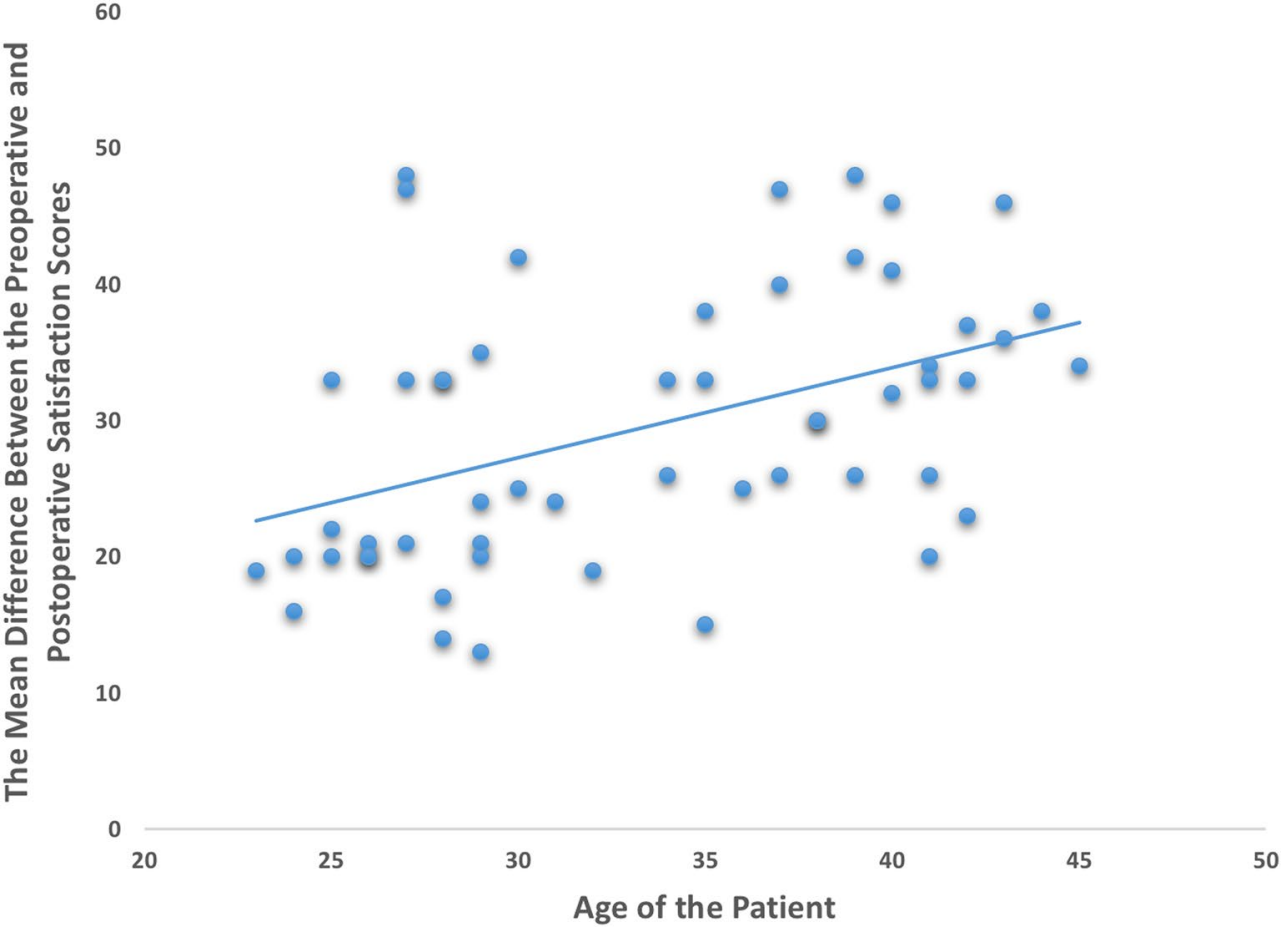
Correlation between the age of the patient and the improvement in satisfaction scores (r = 0.569, *p* < 0.01)

We found that age was a factor which affected the improvements in ROE satisfaction scores after the revision. There was a significant positive correlation between the age of the patient and the improvement in satisfaction scores (r = 0.569, *p* < 0.01) (Fig. 2). In other words, younger patients tend to have less satisfaction increment after the surgery.

**Fig. 3:**
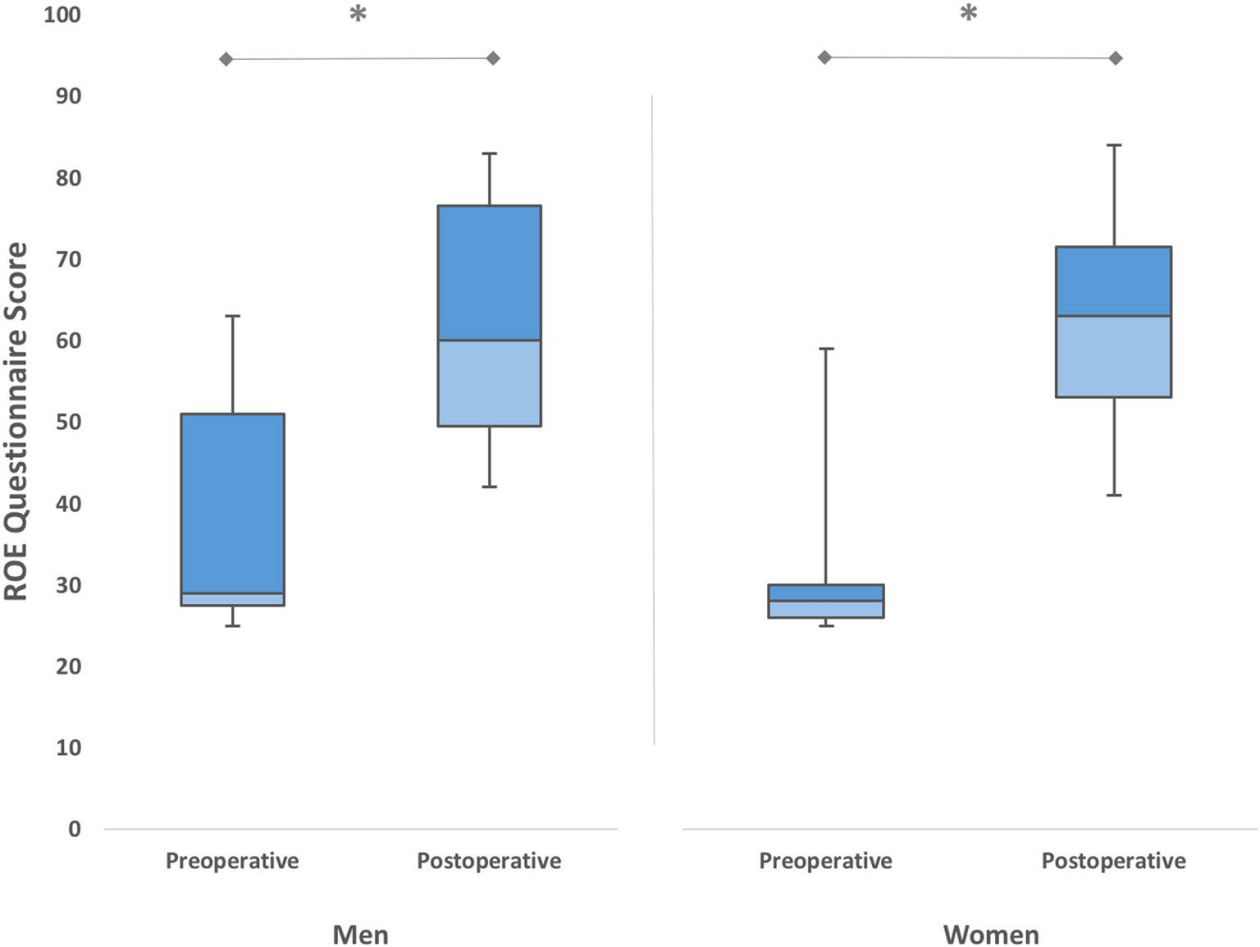
The mean preoperative and postoperative satisfaction scores of patients who underwent revision rhinoplasty, according to the gender (*p < 0.05)

Both male and female patients experienced significant increment in satisfaction scores after the revision (*p* < 0.05) (Fig. 3). We noticed larger differences between preoperative and postoperative ROE satisfaction scores in the women (31.94 ± 10.36) compared with the men (25.26 ± 7.06) (*p* < 0.05). This shows that female patients were significantly more likely to be satisfied after the revision than were male patients.

**Fig. 4:**
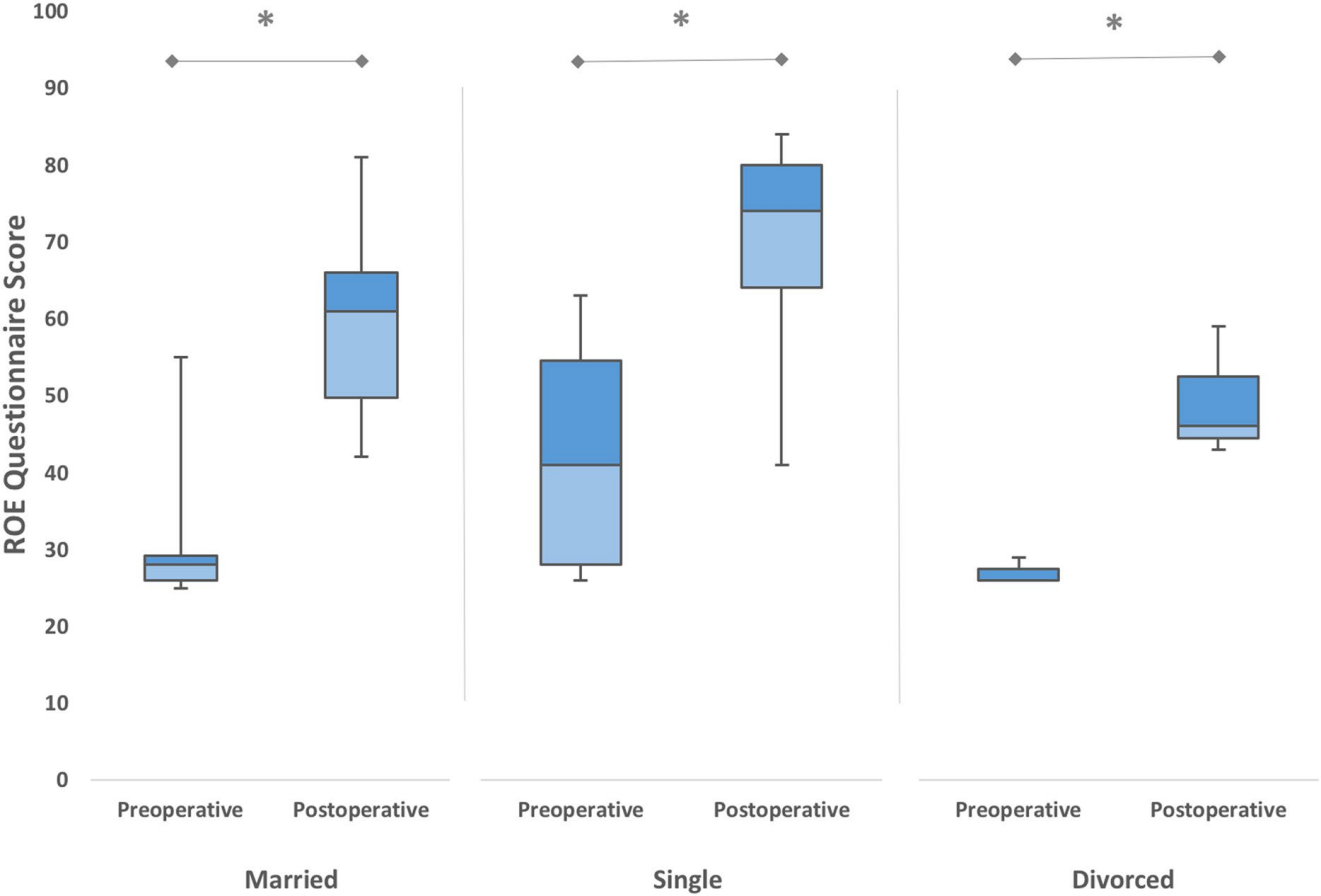
The mean preoperative and postoperative satisfaction scores of patients who underwent revision rhinoplasty, according to the marital status (*p < 0.05)

In our revision rhinoplasty group, 32 patients (59 %) were married, 3 (6 %) were divorced and 19 (35 %) had never been married. According to our results, all three groups experienced significant improvements in satisfaction scores after revision rhinoplasty (*p* < 0.05) (Fig. 4). No statistically significant relationship was observed (*p* = 0.177) in mean differences between preoperative and postoperative satisfaction scores according to the marital status of the patient.

**Fig. 5:**
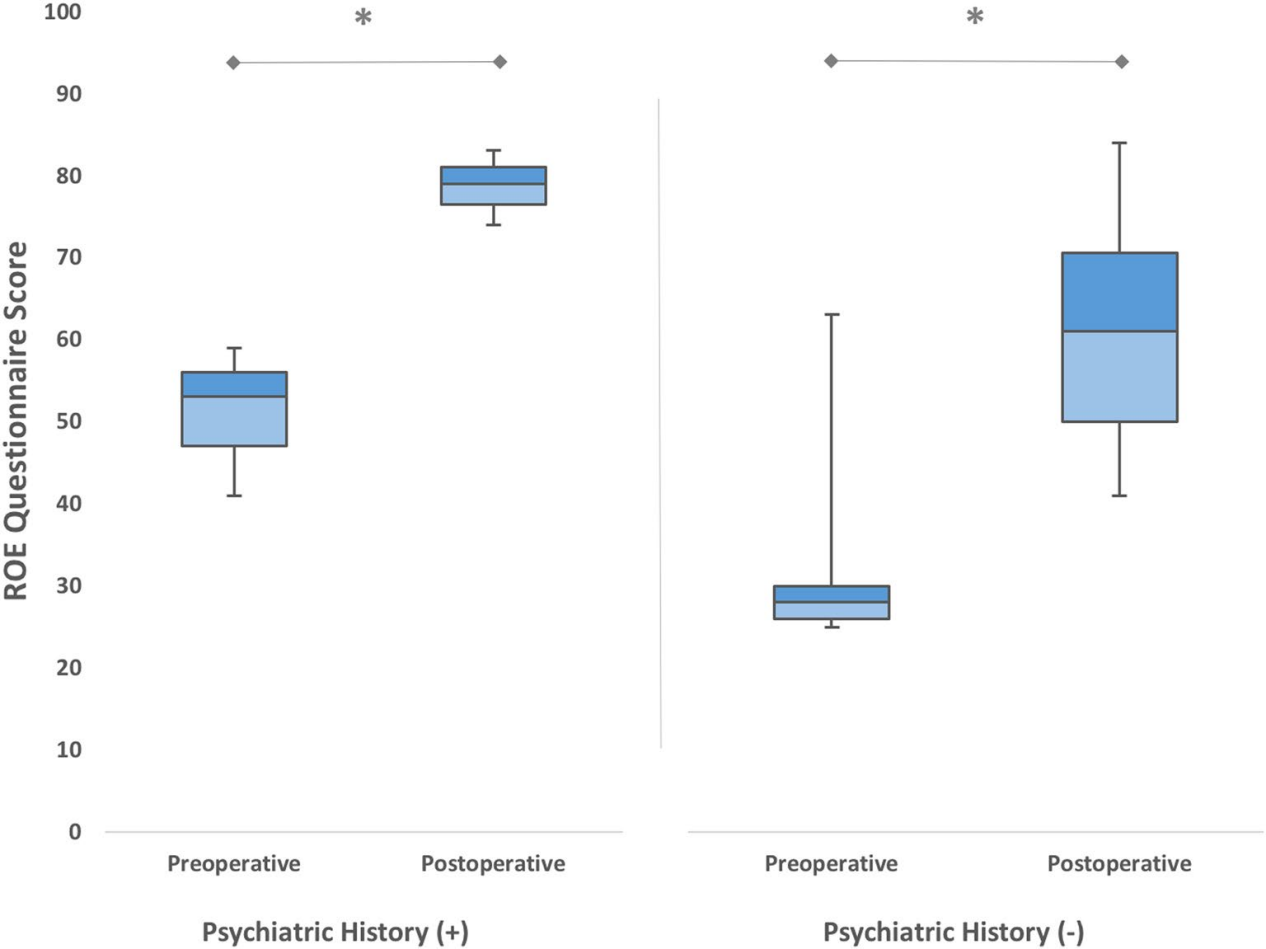
The mean preoperative and postoperative satisfaction scores of patients who underwent revision rhinoplasty, according to the presence of psychiatric history (*p < 0.05)

Patients of the revision group were subdivided into two groups according to presence of a psychiatric history. 3 patients (5 %) were positive for psychiatric history with the diagnosis of depression. Both groups showed significant improvements in ROE scores after the surgery (*p* < 0.05) (Fig. 5). The mean difference between preoperative and postoperative satisfaction scores for patients with psychiatric history was 27.66 ± 4.72 while for patients no psychiatric history it was 29.70 ± 10.04, without any statistically significant difference (*p* = 0.192).

### ROE questionnaire satisfaction scores in relation to the follow-up time

The mean follow-up times after surgery were 13 months (range, 6–23 months) and 11 months (range, 6–17 months) in the revision rhinoplasty and primary rhinoplasty groups respectively. In revision rhinoplasty patients, we found no significant correlation between the gain in ROE satisfaction scores and the follow-up time (*p* = 0.240).

### ROE questionnaire satisfaction scores in relation to the indication for revision

**Fig. 6:**
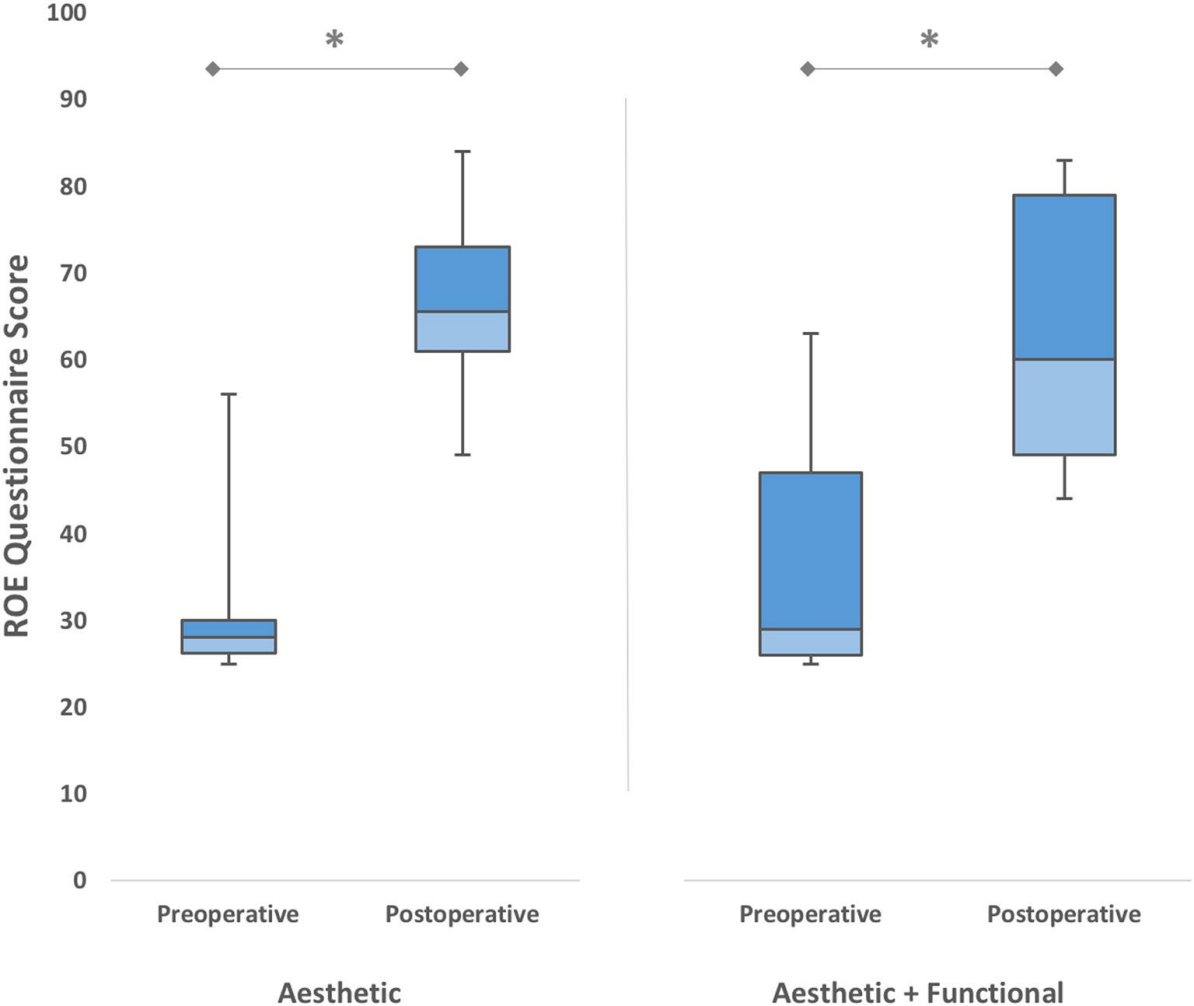
The mean preoperative and postoperative satisfaction scores of patients who underwent revision rhinoplasty, according to the indication for revision surgery (*p < 0.001)

Revision rhinoplasty patients were subdivided into 2 groups according to the reason for undergoing revision. Of the 54 revision surgeries, 30 (55 %) were performed for only aesthetic reasons and 24 (45 %) for a combination of both aesthetic and functional reasons. Both groups experienced significant increments in satisfaction scores by revision rhinoplasty (*p* < 0.001) (Fig. 6). We found that those patients who underwent revision for aesthetic reasons only had significantly higher improvements in ROE satisfaction scores (34.36 ± 9.52) when compared to those patients who underwent revision surgery for a combination of both functional and aesthetic reasons (25.58 ± 6.18) (*p* < 0.01).

### ROE questionnaire satisfaction scores in relation to the graft material used

**Fig. 7:**
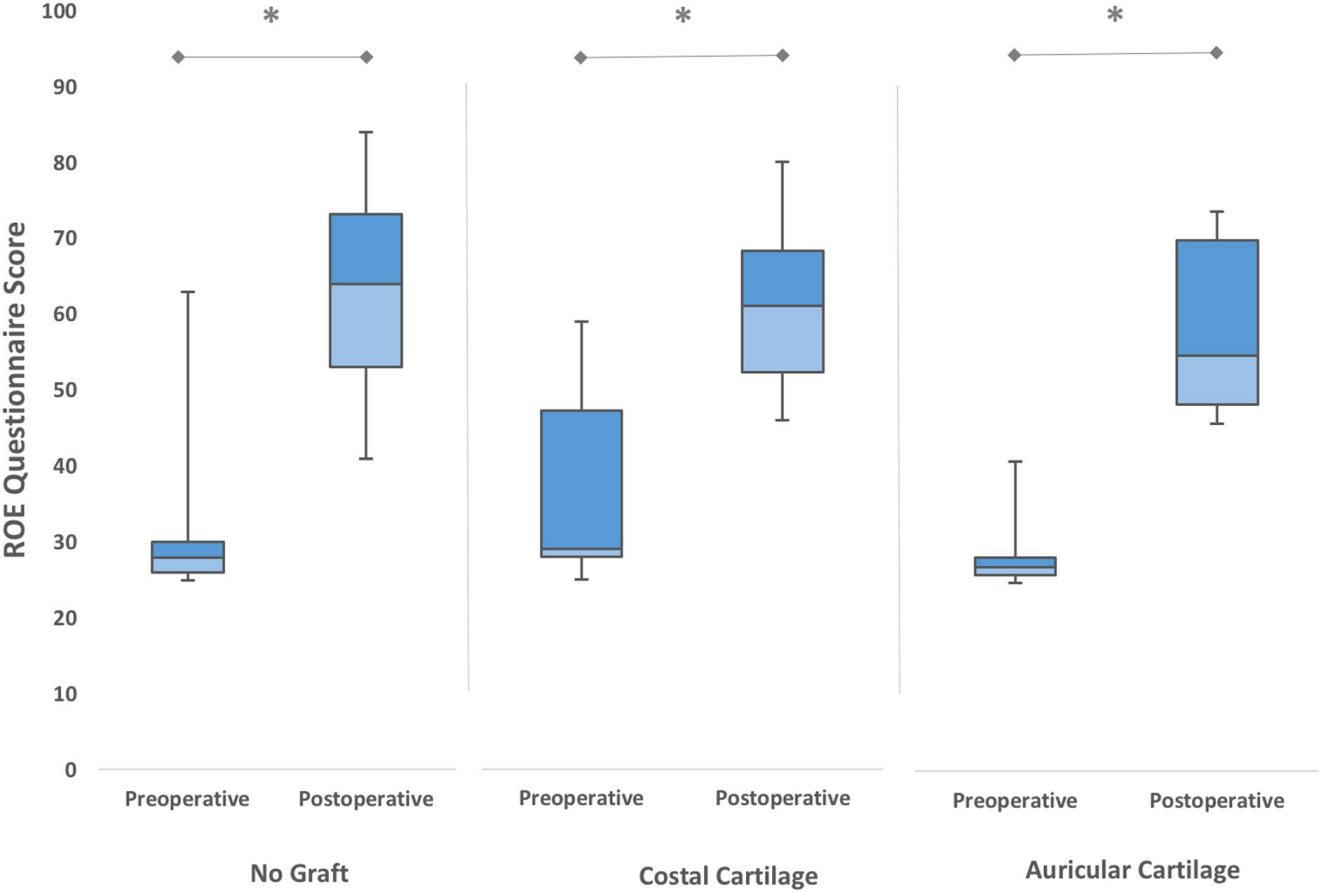
The mean preoperative and postoperative satisfaction scores of patients who underwent revision rhinoplasty, according to the graft material used (*p < 0.01)

In 18 patients (34 %), grafting material had been used in revision surgery (auricular cartilage in 8 patients and costal cartilage in 10 patients). Both grafted and non-grafted patients showed a significant increase in ROE scores after the revision rhinoplasty (*p* < 0.01) (Fig. 7). The gain in ROE satisfaction scores for grafted patients was 27.50 ± 8.42 while for non-grafted patients it was 30.63 ± 10.38, without any significant difference between two groups (*p* = 0.286). In grafted patients, the improvements in ROE satisfaction scores after surgery were irrespective of the graft material used (*p* = 0.515).

### ROE questionnaire satisfaction scores in relation to the satisfaction with the given information and provided care

**Fig. 8:**
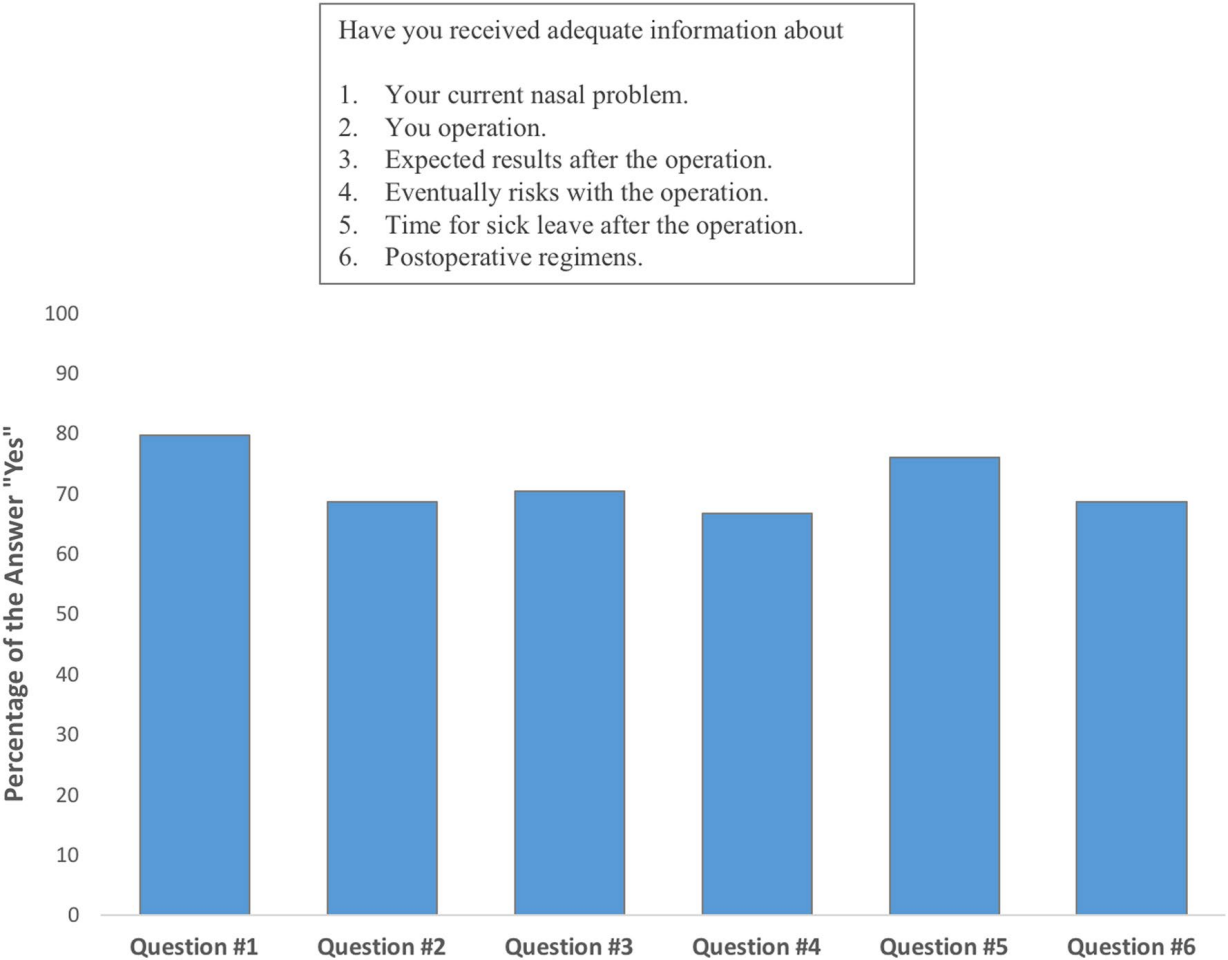
Patients’ answers about the degree of satisfaction with the information given before the surgery

**Fig. 9:**
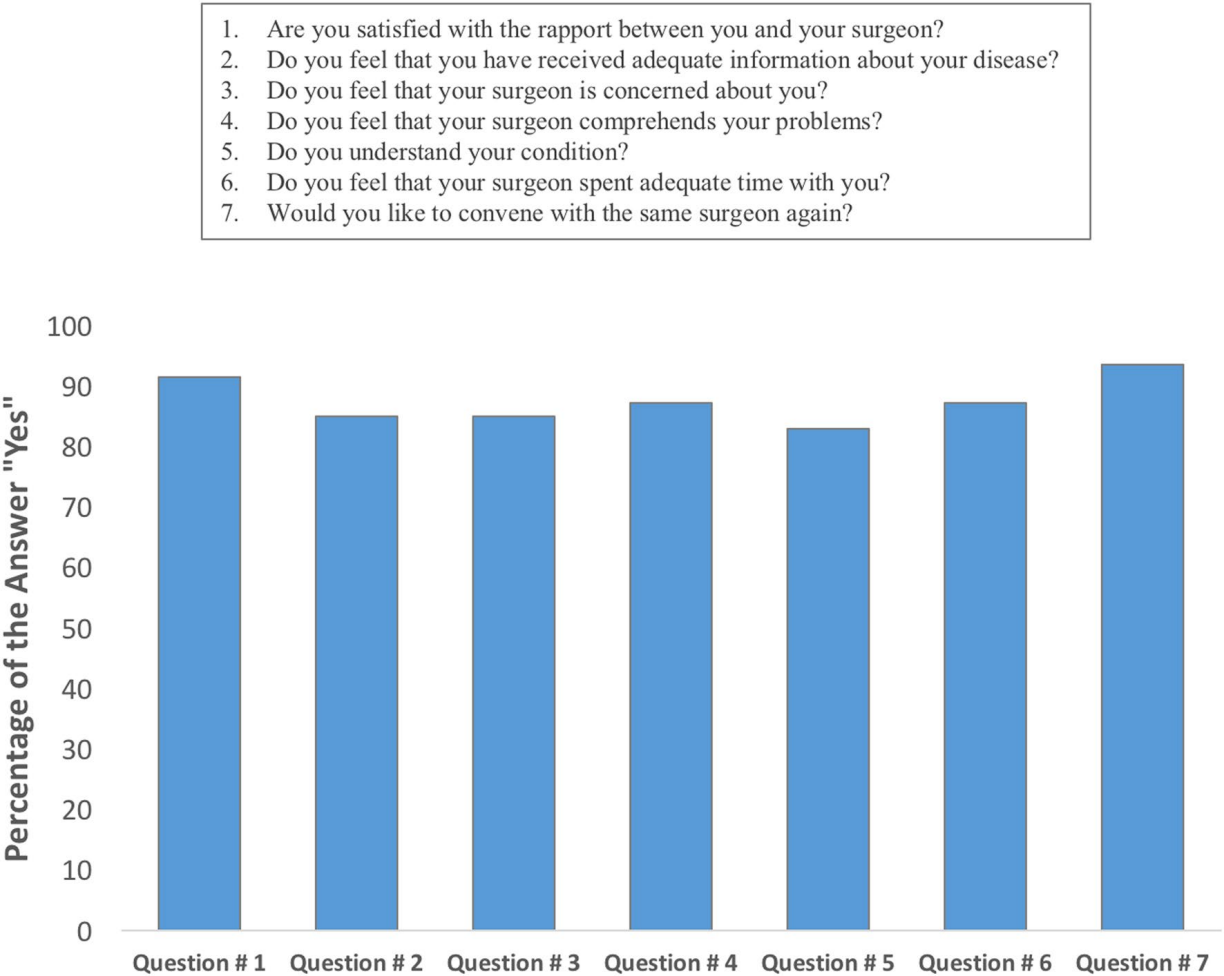
Patients’ answers about the degree of satisfaction with the provided care

**Fig. 10:**
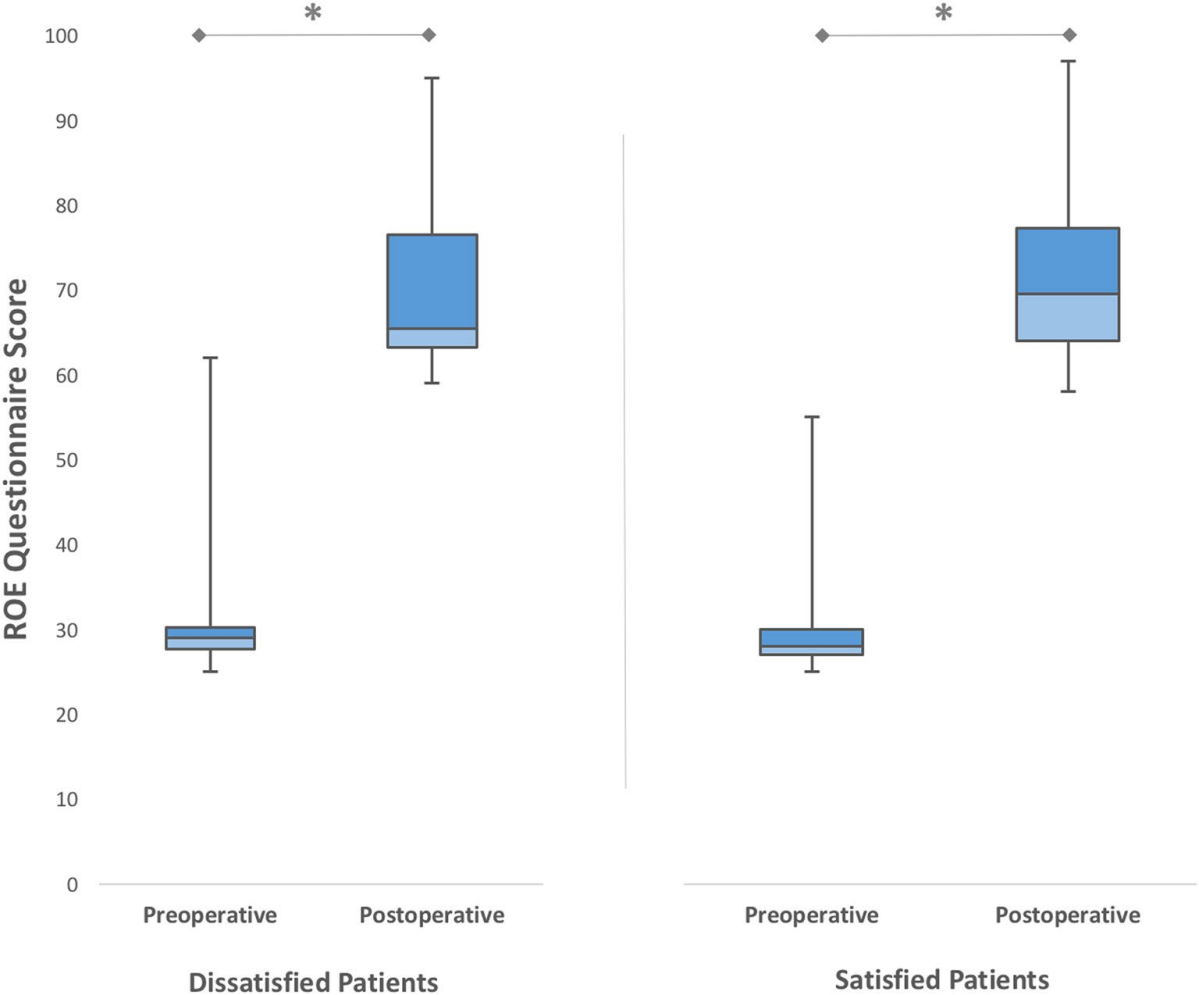
The mean preoperative and postoperative satisfaction scores of patients who underwent revision rhinoplasty, according to the degree of satisfaction with the information given before the surgery (*p < 0.001)

**Fig. 11:**
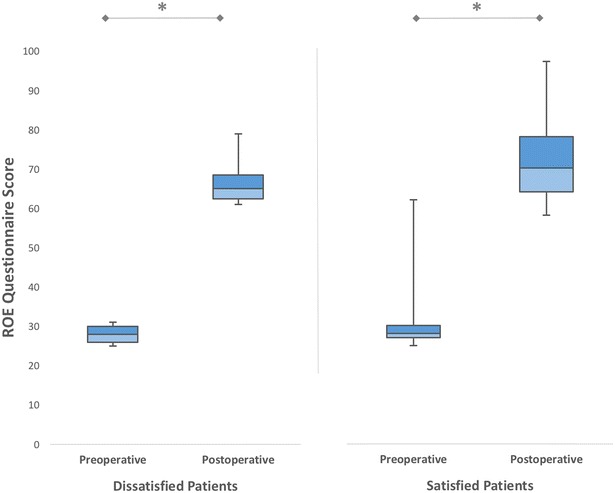
The mean preoperative and postoperative satisfaction scores of patients who underwent revision rhinoplasty, according to the degree of satisfaction with the provided care (*p < 0.001)

The answers for each question about satisfaction with the information given before surgery and provided care are shown in Figs. 8 and 9, respectively. Of the 54 patients, 38 (70.37 %) were satisfied with the given information. Both satisfied and dissatisfied patients experienced significant increments in ROE satisfaction scores (*p* < 0.01) (Fig. 10). The mean improvement in ROE satisfaction scores in patients who were satisfied with the given information (41.57 ± 8.04) was significantly higher in comparison with dissatisfied patients (36.87 ± 5.54) (*p* < 0.001). On the other hand, 47 (87.03 %) patients were found to be satisfied with the provided care. Both satisfied and dissatisfied patients experienced significant increments in ROE satisfaction scores (*p* < 0.001) (Fig. 11). However, we found no significant difference between satisfied and dissatisfied patients in terms of improvements in ROE satisfaction scores (40.40 ± 8.04 and 38.71 ± 4.30 respectively) (*p* = 0.316).

### ROE questionnaire satisfaction scores in relation to the objective evaluation of the nasal deformity

We found no significant correlation between improvements in ROE satisfaction scores after the revision and the results of objective evaluation of nasal deformity in the preoperative setting (Spearman rho = 0.057, *p* = 0.466).

## Discussion

In this study, we demonstrated that all patients underwent revision rhinoplasty experienced a significant degree of satisfaction. The mean difference between preoperative and postoperative ROE satisfaction scores was 29.59, which is higher than the 16 outcome achieved by Hellings and Nolst Trenite (2007) (42.8 before surgery and 58.8 after surgery). We think that the biggest differences between the preoperative and postoperative scores in our study are associated with the shorter mean follow-up time (13 vs. 30 months). Longer follow-up periods may be associated with late complications such as graft loss, nasal collapse, synechiae, and so on (Thomas and Tardy 1986). However, we found no significant relationship between the follow-up period and the satisfaction scores in line with the findings of Arima et al. (2011, 2012).

We found that the improvement in ROE satisfaction scores was significantly higher in primary rhinoplasty patients in comparison with revision patients. Taking into consideration the inherent technical difficulties of revision rhinoplasty, the satisfaction scores of the revision group can be considered high, and even comparable with those of primary rhinoplasty patients.

We documented that younger patients tend to have less satisfaction increment after the revision. This is because these patients may have higher expectations as regards to their postoperative results (Arima et al. 2012). For this reason, we think that this group of patients need to be evaluated more comprehensively in preoperative setting, with more detailed information about the limitations of the surgery.

In this study, we found that male patients were significantly more likely to be dissatisfied than were female patients in agreement with the findings of Gorney and Martello (1999) and Khansa et al. (2016). It has been shown that male rhinoplasty patients have a poorer understanding of their deformity than do women (Wright 1987). In addition, men generally tend to have difficulties in verbalizing the morphologic or functional reasons for their dissatisfaction with the results (Khansa et al. 2016). These findings make it even more significant that the surgeon determines the male patient’s expectations and establish whether they are realistic during the preoperative consultation.

In this study, we found no relationship between the improvements in ROE satisfaction scores and the presence of mental illness. We believe that this finding is associated with low rate of psychiatric comorbidity in our patients. In the revision group, only 3 patients (5 %) were found to be positive for a mental illness. This rate is much lower than the previously reported rates (20–48 %) (Ishigooka et al. 1998; Sarwer et al. 2004).

Whatever the indication for surgery (aesthetic or combination of aesthetic and functional reasons), revision rhinoplasty increased patient satisfaction with highest scores obtained in patients with only aesthetic demands. Actually, we think that such a distinction is absolutely artificial because the aesthetics and function of the nose are inseparably related (Hahn and Becker 2014). For example, aesthetic complaints such as narrowing of the middle vault will generally present concurrently with obstructive symptoms. In this study, we excluded patients seeking revision for only functional reasons because the ROE questionnaire evaluates mainly the aesthetic aspects of rhinoplasty.

In revision rhinoplasty, the use of grafts is indispensable when large amounts of tissue are required (Bussi et al. 2013). Auricular or costal cartilage grafts had been used in nearly one-third of our revision patients. The improvements in ROE satisfaction scores were similar in both grafted and non-grafted patients. Considering that grafting is generally needed in severe nasal deformities, the relatively high rates of satisfaction in grafted patients indicate the functional and cosmetic benefits of grafting. For this reason, surgeons should not hesitate to use grafts if needed. However, we think that longer follow-up time periods are needed to determine the actual satisfaction state in grafted patients.

In the current study, approximately two-thirds of the revision patients reported satisfaction with the information they received in the preoperative visit. This situation was associated with higher rates of satisfaction with the postoperative results. We believe that providing patients with a satisfactory degree of information about the goals, limitations and possible complications of the surgery is fundamental for the exploration of expectations, motivations and perceptions. This is an important function in identifying patients who would benefit from the revision. On the other hand, we did not find any significant relation between the degree of satisfaction with the provided care and the increments in ROE satisfaction scores, which may be attributed to the low percentage of dissatisfied patients with the provided care.

We found no correlation between the severity of preoperative nasal deformity, measured by the subjective evaluation of nasal shape, and the improvements in satisfaction scores. For this reason, we think that even minor nasal deformities should be dealt with seriousness.

This study has some limitations which have to be pointed out. First, such surgeon-initiated questionnaires can be biased in favor of the surgery, because patients may be reluctant to express their dissatisfaction to their surgeons (Lee and Most 2016). We attempted to reduce this bias by asking patients to complete the ROE questionnaire anonymously. We think that the best way to increase the objectivity of such a patient reported outcome study is to conduct the survey by an independent researcher other than the operating surgeon. Second, single-center studies frequently lack the external validity required to generalize the results to a broader population. This is because operative techniques and follow-up protocols after revision rhinoplasty vary widely among surgeons. However, our findings represent a starting point for the evaluation of patient satisfaction after revision rhinoplasty. They allow larger controlled multi-center studies to be planned appropriately in order to include a wider range of population groups and to compare results among centers, all of which increase the generalizability of the results. Third, the design of this study did not allow us to assess the psychological factors that may have an influence on patient satisfaction. However, previous studies showed that the best candidates for rhinoplasty are psychologically stable patients who have requests focused on a specific physical feature (Vuyk and Zijlker 1995). Fourth, the six patients who were called but did not agree to participate in the study could have affected the average gain in satisfaction scores. These patients may not be interested in further evaluation because of their satisfaction or dissatisfaction with the results.

## Conclusion

Using the validated ROE questionnaire, we showed that all patients underwent revision rhinoplasty experienced a significant degree of satisfaction with scores comparable to those of the primary rhinoplasty patients. Our data highlight the importance of the informed consent process when planning revision rhinoplasty, especially on young and male patients.

## Additional files

10.1186/s40064-016-3166-5 Rhinoplasty Outcome Evaluation (ROE) Questionnaire.
10.1186/s40064-016-3166-5 Evaluation of patient satisfaction with the given information.
10.1186/s40064-016-3166-5 Evaluation of patient satisfaction with the provided care.
