# Supplementary material for: Revision rhinoplasty: measurement of patient-reported outcomes and analysis of predictive factors
Source: Springerplus. 2016 Sep 1;5(1):1472. doi: 10.1186/s40064-016-3166-5 (PMC5009061; doi:10.1186/s40064-016-3166-5)
Supplement: Supplementary file 2 — 10.1186/s40064-016-3166-5 Evaluation of patient satisfaction with the given information. [file 40064_2016_3166_MOESM2_ESM.docx]

 **Additional file 2. Evaluation of patient satisfaction with the given information.**
